# Seasonal variations of functional connectivity of human brains

**DOI:** 10.1038/s41598-023-43152-4

**Published:** 2023-10-06

**Authors:** Lyuan Xu, Soyoung Choi, Yu Zhao, Muwei Li, Baxter P. Rogers, Adam Anderson, John C. Gore, Yurui Gao, Zhaohua Ding

**Affiliations:** 1 Vanderbilt University Institute of Imaging Science, Vanderbilt University Medical Center, Nashville, TN USA; 2 Department of Electrical and Computer Engineering, Vanderbilt University, Nashville, TN USA; 3 Department of Radiology and Radiological Sciences, Vanderbilt University Medical Center, Nashville, TN USA; 4 Department of Biomedical Engineering, Vanderbilt University, Nashville, TN USA; 5 Department of Psychiatry and Behavioral Sciences, Vanderbilt University Medical Center, Nashville, TN USA

**Keywords:** Neuroscience, Biomedical engineering

## Abstract

Seasonal variations have long been observed in various aspects of human life. While there is an abundance of research that has characterized seasonality effects in, for example, cognition, mood, and behavior, including studies of underlying biophysical mechanisms, direct measurements of seasonal variations of brain functional activities have not gained wide attention. We have quantified seasonal effects on functional connectivity as derived from MRI scans. A cohort of healthy human subjects was divided into four groups based on the seasons of their scanning dates as documented in the image database of the Human Connectome Project. Sinusoidal functions were used as regressors to determine whether there were significant seasonal variations in measures of brain activities. We began with the analysis of seasonal variations of the fractional amplitudes of low frequency fluctuations of regional functional signals, followed by the seasonal variations of functional connectivity in both global- and network-level. Furthermore, relevant environmental factors, including average temperature and daylength, were found to be significantly associated with brain functional activities, which may explain how the observed seasonal fluctuations arise. Finally, topological properties of the brain functional network also showed significant variations across seasons. All the observations accumulated revealed seasonality effects of human brain activities in a resting-state, which may have important practical implications for neuroimaging research.

## Introduction

It has been long recognized that the mood, cognition, and diverse behaviors of human beings exhibit seasonal variations^1^, a natural phenomenon that can be observed in a substantial portion of the general population^2^. This seasonality exhibits a continuous spectrum of manifestations that range from insignificant to a clinical diagnosis of seasonal affective disorder (SAD)^1,3,4^, between which various levels of seasonal effects may be detected that are below the criteria for clinical diagnosis^5^, and which typically occur during certain months of the year^6–8^.

To understand the physiological mechanisms that underlie seasonal effects, a large body of literature has examined seasonal variations of hormone levels^9,10^, neural transmitter activities^11–14^ and gene expression profiles^15,16^. A recent finding demonstrated that multiple gene expressions across different geographical and ethnic groups showed significant seasonal variations, especially in the immune system^16^. Furthermore, various reports have also characterized the relations between the seasonal variations of mood, cognition and behavioral or physiological variables^17–22^. It has also been shown that changes in photoperiod (daylength) in mammals are encoded by the brain’s central circadian pacemaker, the suprachiasmatic nucleus (SCN)^23,24^, causes changes in melatonin, cortisol and serotonin activity in the brain and subsequently regulates the photoperiodic programming of the excitability of the related neurons^25^. McMahon, et al. suggested that patients with SAD developed depressive symptoms during winter due to an inability to adjust the regulation of serotonin transporters to shorter photoperiods^12^. Further evidence of the presence of seasonality effects on the brain have been shown by measuring seasonal variations of brain electrophysiological activities using electroencephalography^26,27^. Still, while we can point to many examples of seasonal effects on the brain, direct measurements of seasonal variations of brain functional activities remain largely elusive.

More recently, functional magnetic resonance imaging (fMRI) studies of the human brain have been performed using resting-state or specifically-designed task paradigms, to demonstrate variations of brain activities over time scales of months to years^28–32^. Task-dependent cognitive activity, such as working memory and attention, can exhibit intrinsic seasonal effects^32^. An extensive longitudinal study on a single human subject over 18-months showed that brain connectivity as measured by fMRI was highly variable across the time period studied, as well as other psychological and biological variables^30^. Di et al. characterized the influence of seasonal correlated environmental factors on brain function using machine learning regression, and showed that several parameters, especially daylength and air temperature, had the highest prediction accuracies^28^. Reproducibility of human fMRI studies is a constant concern. An understanding of the magnitude and pattern of seasonality effects that may confound normal variations of human brain activity detectable by fMRI will be important for neuroimaging and cognitive neuroscience research moving forward.

Previous research has shown that white matter (WM) encodes important spatiotemporal information^33–35^ and WM functional networks demonstrate stronger dynamic connectivity than gray matter (GM) functional networks^36^. Patterns of connectivity between WM and GM have been observed giving support to the idea that BOLD signals in WM are related to neural activities in the brain^37^. Synchronous neural activity between GM regions and WM bundles can be summarized in functional connectivity (FC) matrices and the global average of GM–WM connectivity can quantify the overall synchronization of functional brain activities^37^.

Given the previous evidence showing physiological and cognitive variations across seasons, we hypothesized that large-scale differential seasonal patterns in brain connectivity captured by fMRI would potentially be observable in typical adults. We began with fractional amplitudes of low-frequency fluctuations (fALFF) of BOLD signals in GM regions and in WM bundles which report the intensity of spontaneous baseline activity and have been suggested to reflect cognitive activity^38^. We then compared measures of FC between GM regions and WM bundles at a global level and then at a network level considering possible effects of seasonality, and we further explored how environmental factors, including average temperature and daylength, affect seasonality effects on resting-fMRI brain connectivity. Lastly, seasonality effects of topological properties of functional brain networks were further characterized.

## Results

Resting-state fMRI data from 410 subjects (female: 247 subjects; male: 163 subjects) obtained through the HCP 3T database were examined. Ages of the participants ranged from 26 to 35 years old. The seasons were divided using the acquisition quarter information provided by the HCP project wiki^39^ and defined as follows: Spring is from February 1st to April 30th; summer is from May 1st to July 31st; autumn is from August 1st to October 31st; and winter is from November 1st to January 31st. The acquisition seasons of the subjects spanned from winter in 2013 (winters included an extra month in the following year) to autumn in 2015, which covered two consecutive years (see Table 1 for the number of subjects analyzed for each season). First, we analyzed global seasonal variations of fALFF in GM and WM, and of FC between GM regions and WM bundles. Correlations between the global activity metrics and environmental factors were further sought. Second, we specifically characterized seasonality effects for 14 large-scale brain networks, with each representing a distinct functional module in the brain. Detailed WM bundles, GM regions (along with their abbreviations), and the list of all functional networks are presented in Supplementary Tables S1 and S2. Finally, seasonal variations of topological properties of the brain networks were quantitatively examined. For all the functional metrics studied, we began with a statistical test of significance of seasonal variations using ANOVA. Then we performed detailed comparisons between distinct seasons for those exhibiting significant seasonal variations and evaluated the degree of periodicity in their seasonality effects by using sinusoidal function fitting.

**Table 1:** Numbers of female and male subjects in each acquisition season from winter in 2013 to autumn in 2015.

	Winter	Spring	Summer	Autumn
No	127	99	91	93
No. of females	78	61	57	51
No. of males	49	38	34	42

### Global seasonal effects

**Figure 1:**
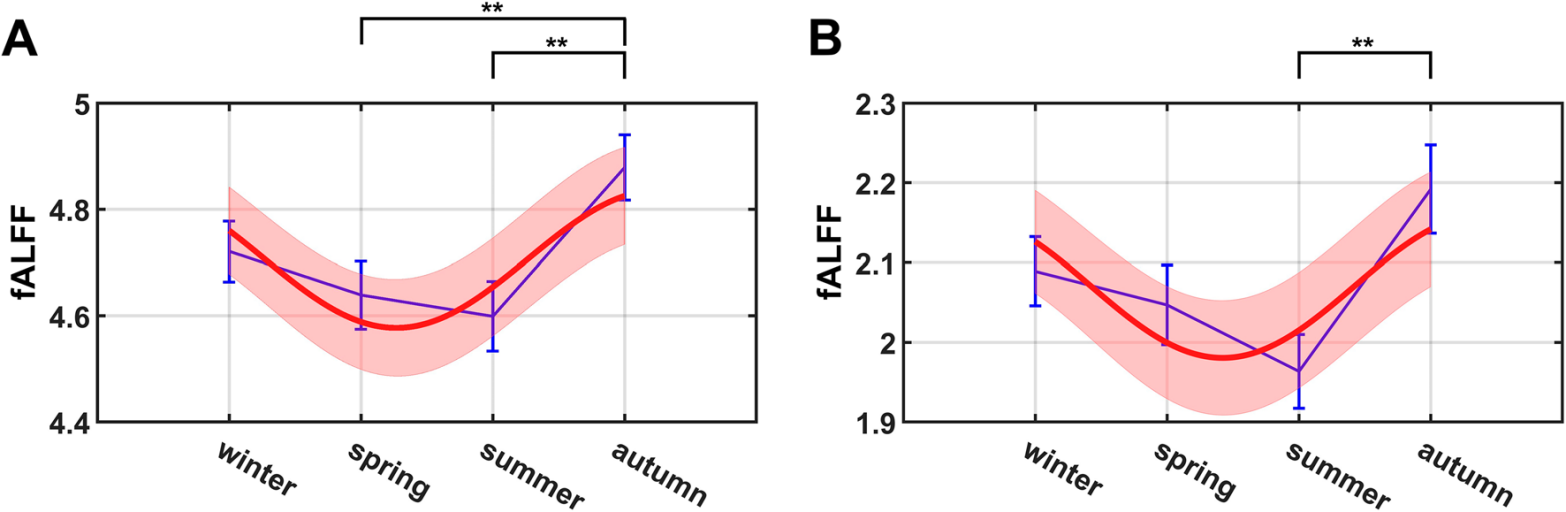
Group mean fALFF (blue) across seasons overlaid with proposed sinusoidal function fitting to test for periodicity and the related interval at 95% confidence level (red) separately for (**A**) GM fALFF and (**B**) WM fALFF. The error bars (blue) are plotted based on the standard errors of the mean in each season. Note: **p < 0.01.

At the global level, we first examined seasonal fluctuations of mean fALFF for GM and WM respectively. Averaged power spectra were computed for each GM region and WM bundle, from which a mean GM and WM fALFF value was obtained for each subject. ANOVA tests showed that the mean fALFF was significantly different across the four seasons ($p<0.05$) for both GM and WM (GM: $p=0.0128$, effect size Cohen’s $f$ (abbreviated as $f$ in the follows) $=0.1644$ ; WM: $p=0.0165$, $f=0.1601$), indicating the existence of seasonal variations of the low-frequency oscillations in BOLD signals in both GM and WM. Overall, the mean fALFF of GM and WM had largely similar patterns of seasonal variations, which gradually decreased from winter to summer followed by increases to a peak value in autumn. Detailed statistical comparisons revealed that there were significant differences in GM fALFF between autumn and spring ($p=0.0076$, effect size Cohen’s $d$ (abbreviated as $d$ in the follows) $=0.3894$) and between autumn and summer ($p=0.0022$, $d=0.4579$); a significant difference in WM fALFF was observed between autumn and summer ($p=0.0019$, $d=0.4651$). The full power spectra of GM and WM for each season are shown in Supplementary Fig. S1 and S2 in our supplementary material. Power spectra were calculated from normalized signals of each GM region and WM tract, which were then averaged over all GM regions and WM tracts separately and across all the subjects for each season. As can be seen, the power within the range of 0.01–0.08 Hz tends to be higher in autumn for both GM and WM than in other seasons, consistent with our findings with respect to fALFF. Evaluations of the degree of periodicity of fALFF seasonality by sinusoidal fitting (with amplitude and phase as variables) are shown in Fig. 1 for GM (1A) and WM (1B) fALFF, in which the fitted fALFF values (red curves) are displayed with their 95% confidence intervals (orange areas). Further F-tests found that the seasonal variations of both GM and WM fALFF could be well modeled by the sinusoidal function (GM: $p=0.0158$, $f=0.1436$; WM: $p=0.0359$, $f=0.1283$), which showed that the fALFF tended to be higher in autumn and winter than in spring and summer.

**Figure 2:**
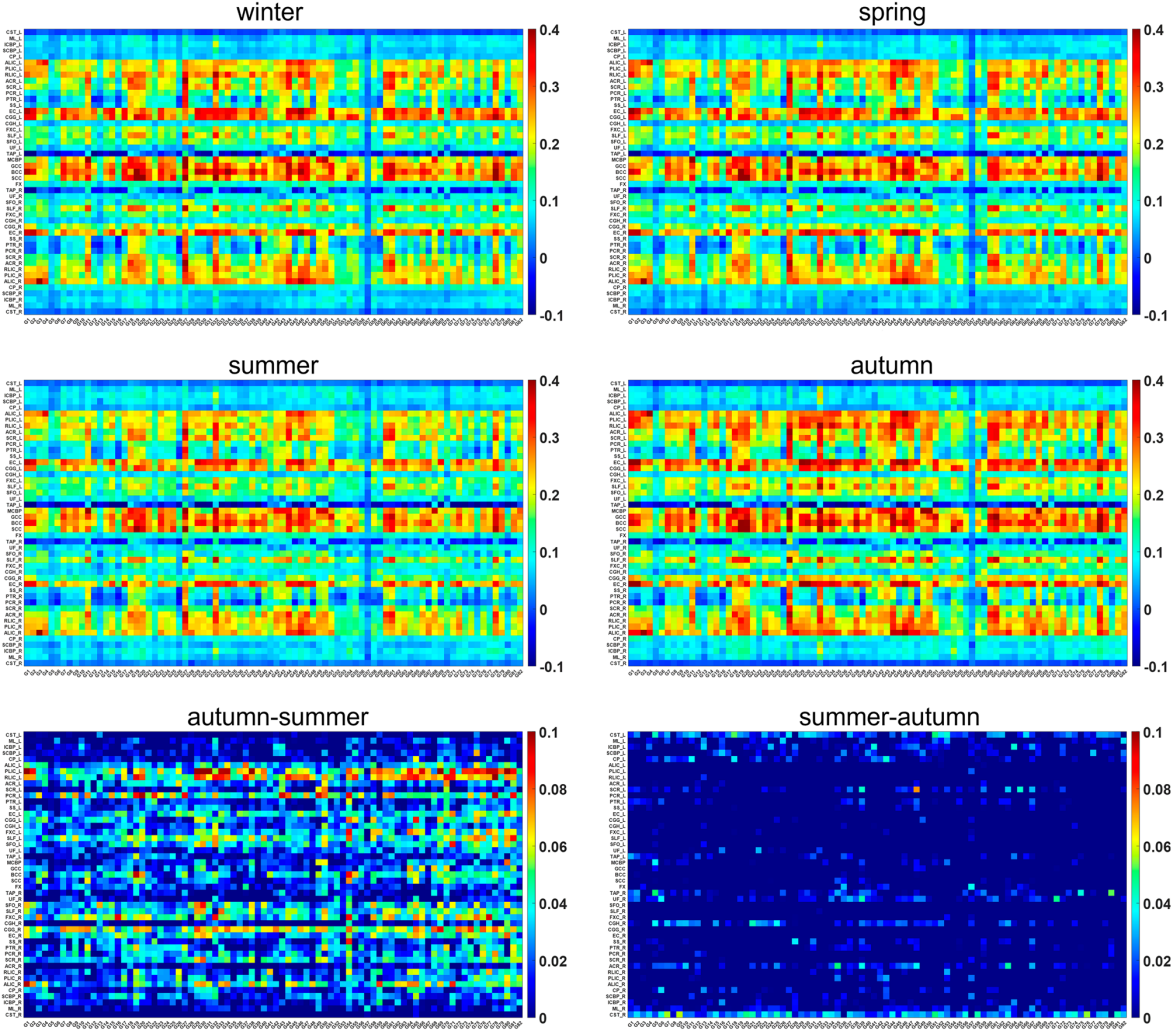
Maps of the group mean GM-WM FC matrix for four seasons and the difference matrices between the autumn and summer season. Overall, the autumn appears to have stronger FC and the summer tends to have weaker FC than other seasons. Note that the lower left image in the bottom row shows the difference between autumn and summer when FC in autumn is greater than summer, and the lower right image shows the difference in the opposite direction.

The above analysis was also applied to the FC between GM regions and WM bundles. First, the coefficient of Pearson correlation was calculated between each pair of GM region and WM bundle, defined respectively by Shirer et al.^40^ and the JHU ICBM-DTI-81 WM atlas^41^, yielding a GM-WM FC matrix for each subject. Figure 2 illustrates the group mean GM-WM FC matrices in four seasons. It appears that the autumn tends to have stronger FC and the summer tends to have weaker FC than other seasons. The detailed difference in the mean FC matrix between these two seasons is also shown in Fig. 2. As expected, it is evident that when compared to the summer season, the group mean GM-WM FC matrix of the autumn season exhibited a higher FC intensity in the majority number of matrix elements.

**Figure 3:**
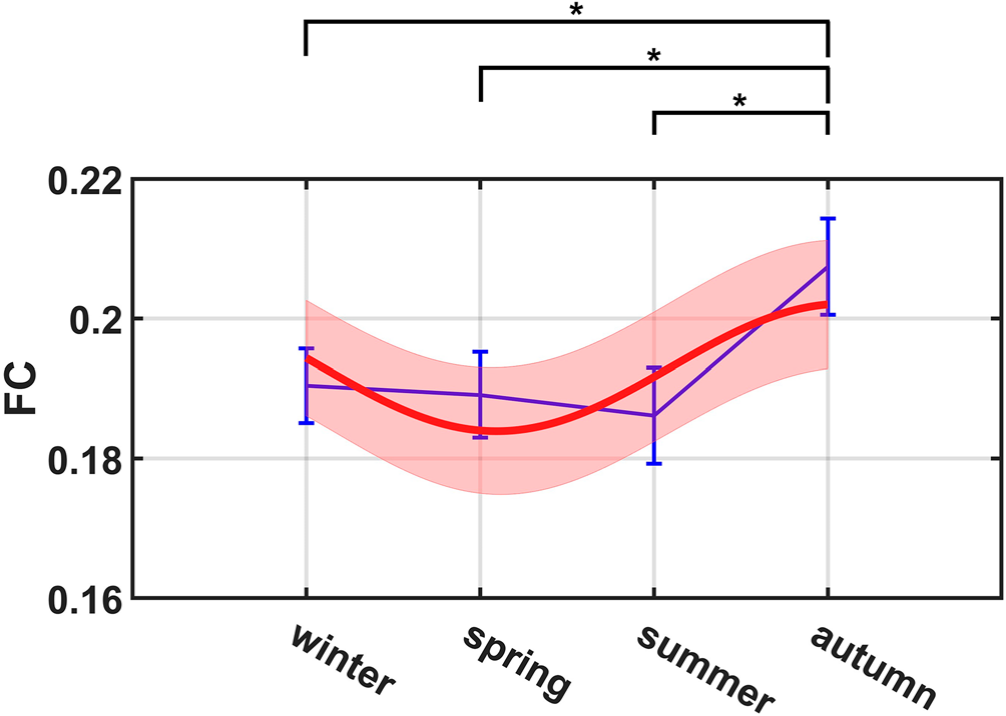
Group mean FC (blue) of GM-WM across seasons overlayed with proposed sinusoidal function fitting to test for periodicity and the related interval at 95% confidence level (red). The error bars (blue) are plotted based on the standard errors of the mean at each season. Note: *p <  0.05.

To further quantify the seasonal variation of GM-WM FC, each correlation coefficient in the FC matrix was then converted to a Z-score by pooling over all the subjects studied and a mean Z-score was obtained for each FC matrix. Our ANOVA tests showed that global FC strength, defined as the grand mean of the mean Z-score over all the subjects in a season group, exhibited relatively less significant variation across the seasons compared to fALFF behaviors ($p=0.0889$, $f=0.1273$), but significant differences were still found between seasons. Global FC strength was found to be highest in autumn and lowest in summer ($p=0.0303$, $d=0.3219$). Significant differences were also observed between autumn and spring ($p=0.0480$, $d=0.2874$) and between autumn and winter ($p=0.0483$, $d=0.2710$), as shown in Fig. 3. Fitting of the global FC strength with a sinusoidal function did not reach the significance level ($p=0.1360$, $f=0.0992$), although the least square fitting showed a peak FC during autumn, just like the fALFF in Fig. 1. Finally for completeness, the mean FC of GM-GM and WM-WM matrix was also analyzed similarly, with results reported in Supplementary Fig. S3. Briefly, the mean WM-WM FC was significantly different across the four seasons ($p=0.0102$, $f=0.1681$) and could be well fitted by a sinusoidal function ($p=0.0488$, $f=0.1221$), whereas seasonal variations of the mean GM-GM FC did not reach the significant level ($p=0.1755$, $f=0.1108$) nor their fitting with a sinusoidal function ($p=0.1070$, $f=0.1050$).

To explore the relationship of the above functional activity and connectivity metrics and environmental factors, average environmental parameters, including average temperature and daylength in St. Louis, Missouri in the years of acquisitions, were used as regressors to predict the mean GM and WM fALFF and global FC strength. Seasonal variations of both the mean GM and WM fALFF could be well predicted by the average temperature and daylength (GM fALFF: $p=0.0121$, $f=0.1479$; WM fALFF: $p=0.0282$, $f=0.1331$), whereas the prediction of global FC strength failed to reach the significance level. Note that a temporal delay was observed between the environmental parameters and GM/WM fALFF (see Supplementary Fig. S4), with the fALFF values peaking in autumn while the average temperature and daylength reaching their peaks in summer.

### Seasonal effects across large-scale brain networks

**Figure 4:**
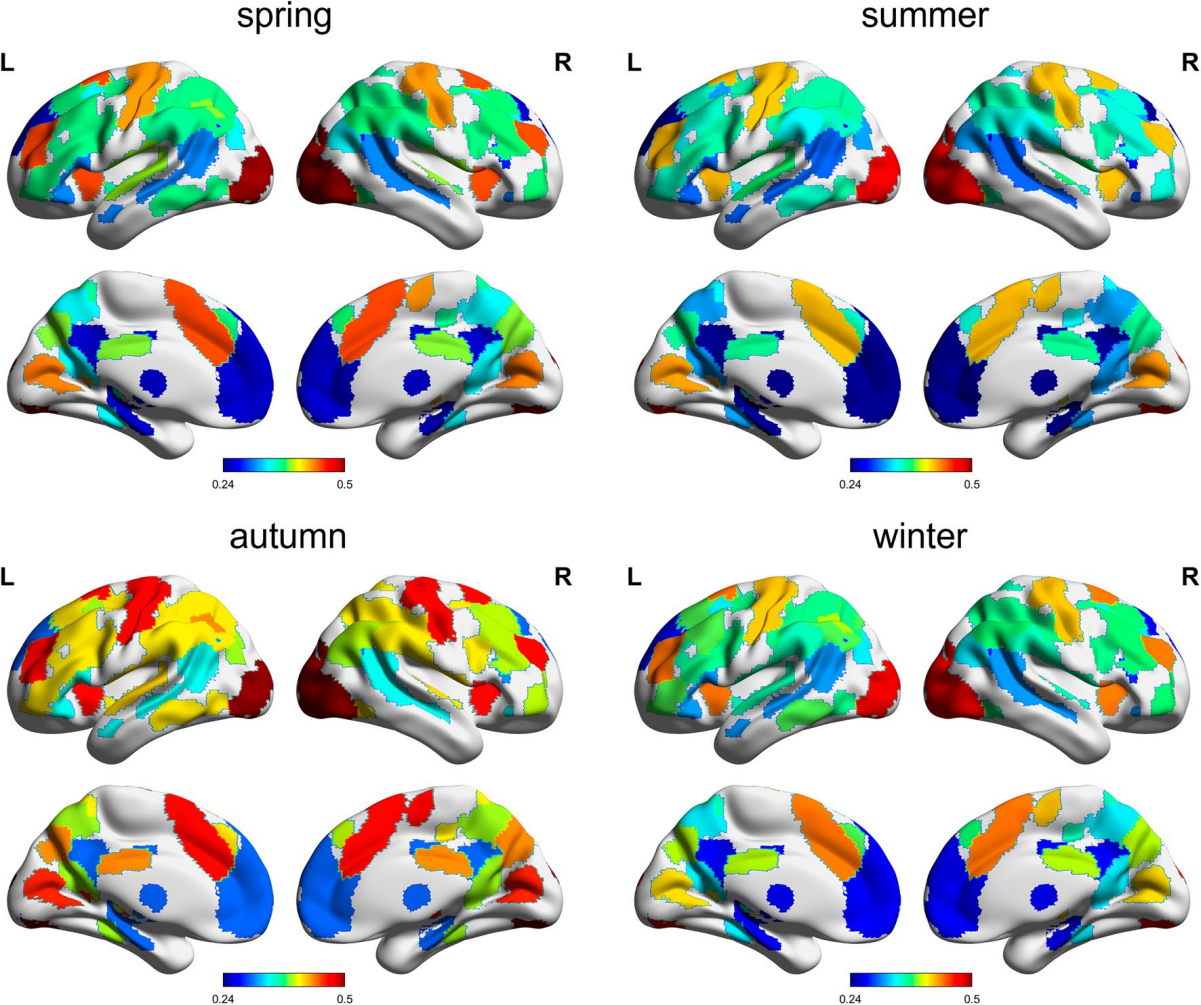
Maps of the WM-averaged FC in grayordinate space in four seasons. The area shown in the figure is covered by the 14 functional networks used, and the FC intensity value is denoted by the color bar provided at the bottom of each map.

**Figure 5:**
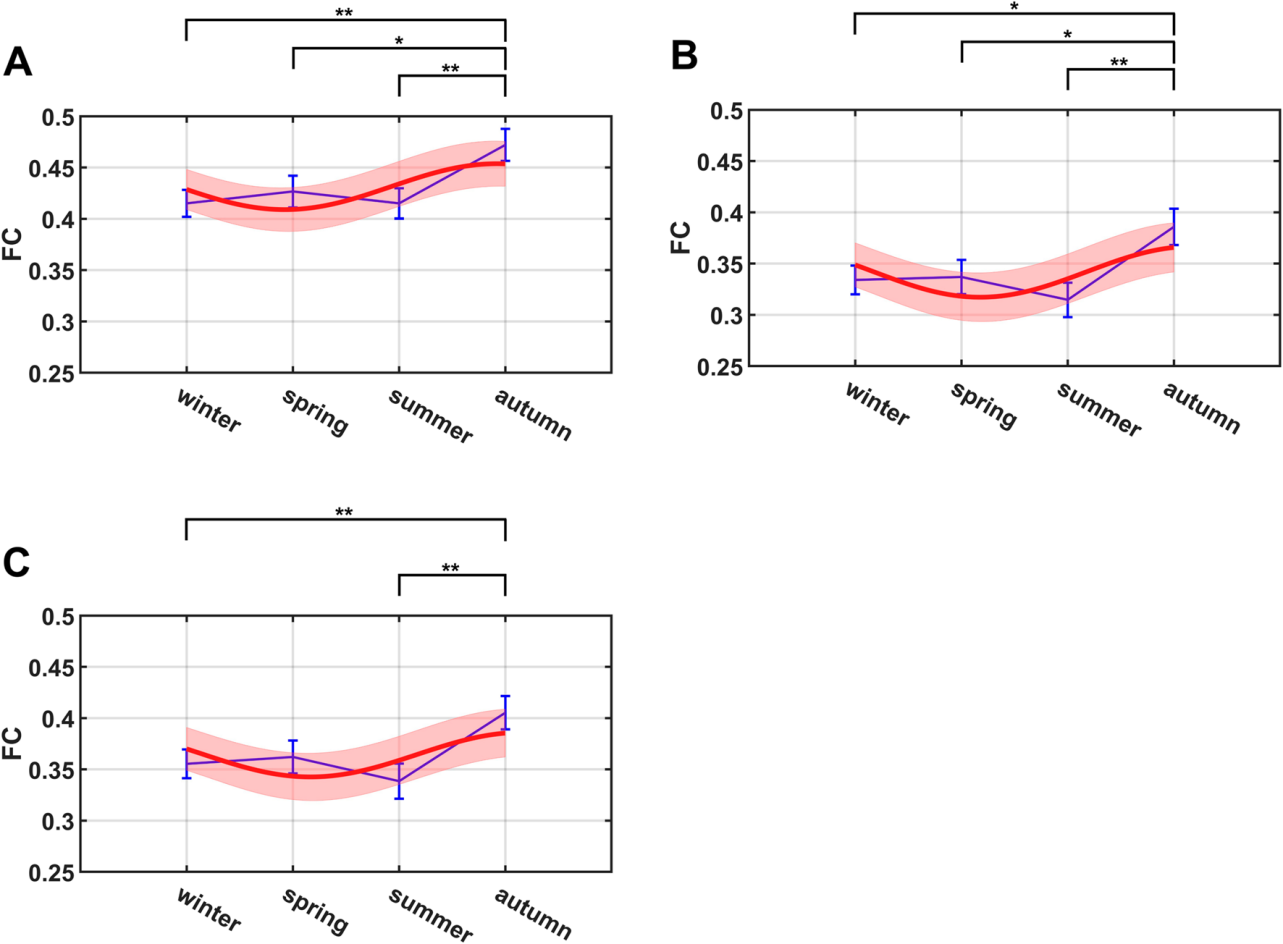
Group mean FC of brain networks (blue) across seasons overlayed with proposed sinusoidal function fitting to test for periodicity and the related interval at 95% confidence level (red) separately for (**A**) sensorimotor, (**B**) ventral DMN, and (**C**) posterior insula networks. The error bars (blue) were plotted based on the standard errors of the mean at each season. Note: *p <  0.05; **p <  0.01.

Seasonal variations were further explored in 14 large-scale brain functional networks defined by Shirer, et al.^40^. Specifically, the GM-WM FC matrix computed above was divided into 14 submatrices to derive a mean Z-score for each of the 14 networks (see Fig. 4 for detailed seasonal variations in grayordinate space). Similar to the global analysis, significant seasonal variations were observed in several functional networks, with ANOVA tests demonstrating that the Z-scored mean FCs of the posterior insula, sensorimotor, and ventral DMN networks were significantly different ($p<0.05$) across the four seasons (posterior insula: $p=0.0305$, $f=0.1491$; sensorimotor: $p=0.0224$, $f=0.1547$; ventral DMN: $p=0.0231$, $f=0.1542$). The FC strength of all the three networks was found to be highest in autumn, which showed significant differences with the other seasons. In this work, the highest FC strength was approximately 20% greater than the lowest, revealing sizeable seasonal FC variations throughout the year. The mean FC of these three networks however did not pass the significance test of periodicity with the sinusoidal model. Detailed statistical comparisons between the four seasons for the above three networks are graphically summarized in Fig. 5. The results of the remaining networks are presented in Supplementary Fig. S5.

### Seasonal variations of network properties

**Figure 6:**
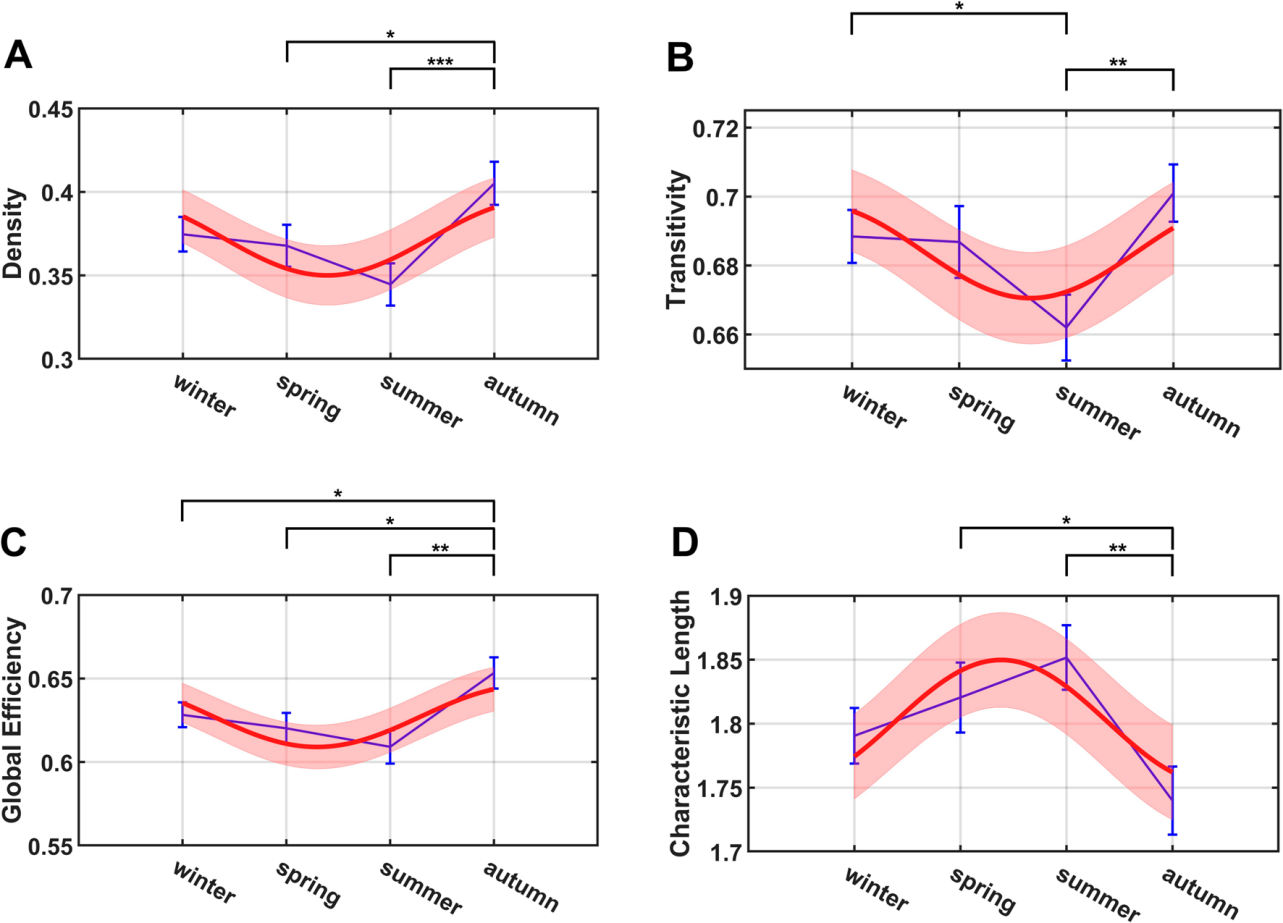
Group mean network properties (blue) across seasons overlayed with proposed sinusoidal function fitting to test for periodicity and the related interval at 95% confidence level (red) respectively for (**A**) density, (**B**) transitivity, (**C**) global efficiency and (**D**) characteristic path length. The error bars (blue) are plotted based on the standard errors of the mean in each season. Note: *p <  0.05; **p <  0.01; ***p <  0.001.

To examine seasonal variations of overall brain functional architecture, particularly seasonal variations of topological properties of the brain networks, we computed a number of graph theoretic parameters for each of the subjects studied and quantified their fluctuations across the four seasons. For each subject, we first calculated GM-GM, WM-WM and overall FC matrices (i.e., (GM ∪ WM)-(GM ∪ WM)), from which density, transitivity, global efficiency, and characteristic path length were derived from each of these FC matrices. Our ANOVA tests found that all the network properties of WM-WM functional connections were significantly different across the four seasons (density: $p=0.0091$, $f=0.1700$; transitivity: $p=0.0305$, $f=0.1491$; global efficiency: $p=0.0071$, $f=0.1740$; characteristic path length: $p=0.0210$, $f=0.1559$). Autumn showed significantly higher density, transitivity and global efficiency, and smaller characteristic path length than the other seasons, a pattern that resembled that of global fALFF and FC. In detail, there was significantly higher density and smaller characteristic path length of WM-WM networks in autumn than in spring and summer, and significantly higher global efficiency in autumn than in the other three seasons. Significant differences in transitivity were also found between autumn and summer and between winter and summer. Further analysis revealed that the density, global efficiency, and characteristic path length of WM-WM networks could be well fitted by sinusoidal functions (density: $p=0.0358$, $f=0.1283$; global efficiency: $p=0.0183$, $f=0.1410$; characteristic path length: $p=0.0286$, $f=0.1327$). The sinusoidal fittings of the topological properties and their 95% confidence levels are shown in Fig. 6. Note that ANOVA tests on these topological properties of GM-GM and overall FC matrices were not significant, although similar trends could still be observed.

## Discussion

We report our preliminary studies exploring seasonal variations in measures of brain activity derived from fMRI. Global- and network-level measures of functional connectivity, low frequency fluctuations of regional functional signals, and topological properties of brain functional networks, all demonstrated significant seasonality effects. In particular, three specific networks, involving sensorimotor, posterior insula, and ventral DMN regions, revealed significant seasonal variations in mean FC strength between seasons. Across our different observations, autumn contrasted most consistently from the other three seasons. We observed higher global- and network-level functional connectivity strengths, as well as WM and GM fALFF values, in autumn. In addition, topological analysis of brain functional networks detected higher connectivity density, transitivity and global efficiency, and lower characteristic path length in autumn as well. We were also able to correlate environmental factors with seasonal brain functional activities. Together, these observations provide evidence of an influence of seasonality effects on physical indices of human brain activities in a resting-state.

Previous research has demonstrated that the overall brain performance reaches its peak around the autumn season, especially the brain activities relevant to cognitive processing^42^ in which the sensorimotor network also plays a significant role^43–45^. This is in line with our observation that GM-WM FC in the sensorimotor network reached the highest point in the autumn. The DMN network, which is believed to be active during a subjects’ internal mental processes^46^, plays an important role in the interactions between other brain systems^47,48^. It has been argued that the DMN is a key contributor to the organization and expression of preplanned, reflexive behaviors^48^. Our work revealed a significant seasonal variation of FC in the ventral DMN, which is significantly higher in autumn than in other seasons, supporting the notion that the DMN might be a driving factor responsible for seasonal variations of brain connectivity at both global- and network-levels. Much similar to the sensorimotor and ventral DMN system, the posterior insula network also exhibited significantly higher FC in autumn. Prior studies have identified seasonal fluctuations in brain responses, some of which peaks in the autumn, particularly that related to insula^32^. This phenomenon is thought to be associated with the variations in brain dopamine concentration, which exhibits elevated levels during the autumn^11,49^. The seasonality effects of GM–WM FC at resting state in the insula we observed could be similarly mediated by dopamine.

It has long been observed that human behaviors, including brain activities, are strongly influenced by the environment where individuals reside^1^. Our analysis of the relationship between the seasonal variations of brain functional measures and local environmental factors showed that multiple covariates, including air temperature and daylength, are closely correlated with the fALFF of GM regions and WM bundles. Light exposure is one of the most common factors related to seasonality, and its intensity and spectral distribution vary with changing seasons^50^. Average temperature is also a strongly entangled factor due to its strong correlation with light duration. Our results show that the fALFF of GM and WM could be influenced by the temperature and daylength as these measures demonstrated similar periodicity. It should be noted that these environmental factors are widely recognized to vary remarkably with geographic locations, which suggests that the seasonality found in this work could differ in other areas. Finally we emphasize that, rather than isolating the subjects from the natural environment prior to the scanning processes as implemented by Meyer et al.^32^, this work made use of data acquired without the isolation of external factors and thus provided a means of quantifying the role of environmental factors on seasonality effects.

Practically, this work potentially has great importance for imaging-based brain research from both clinical and technical perspectives. First, our observation that brain functional activities vary seasonally at both the global- and network-level can be used to guide optimal treatment for clinical populations with abnormalities paced by the season, especially the spectrum of SAD. The sensorimotor network is found to be overactive in SAD patients^51^, and thus can be chosen as a target of their medical treatments or physical therapies. Alternatively, the ventral DMN region or perhaps even more effectively the posterior insula network can be modulated to ameliorate the SAD symptoms. Second, our finding that the magnitude of seasonal variations of brain activities can be, somewhat surprisingly, as large as those induced by many neurological conditions may have important technical implications for clinical neuroimaging experiments. Specifically, to minimize the impact of seasonal factors on the main effect of interest, precautions must be exercised in designing functional neuroimaging experiments as well as in analyzing and interpreting the data, such that environmental parameters at the time of image acquisitions are appropriately considered or controlled.

Our findings revealed significant differences between brain activities in distinct seasons, and significant seasonal variations, which could be well fitted by sinusoidal functions. A recent study drew extensive attention when it illustrated poor test–retest reproducibility of fMRI measurements^52^, raising the awareness of reliability issues in current fMRI procedures. This study repeated scans approximately five months apart, during which seasonal environmental parameters can introduce substantial changes. It should be pointed out that the stability of the scanning instruments used in the procedure of this work was not examined due to unavailability of calibration data. However, we can reasonably assume that potential seasonal variations of instruments do not dominate our experimental findings as our imaging effects are not general and the data used are essentially normalized relative to a baseline. Furthermore, we divided the subjects into female and male groups. It was found that the two groups exhibit quite different seasonal patterns (particularly in GM-WM FC), with the female group experiencing significant seasonal difference between the winter and autumn seasons while no significant differences were observed between any two seasons for the male group. Also, it was found that there existed significant difference between the male and female groups for the winter season, as shown in Supplementary Fig. S6. The sex-dependent patterns of seasonal variations thus have basically ruled out that the observed seasonality effects are primarily driven by the scanning instruments.

Another potential limitation of this work is the lack of detailed acquisition dates. Female and male subjects were grouped based on their acquisition seasons and each season was treated as one time point for the fitting of seasonal variations. Of particular note, the seasonal variations observed in this work were fitted using sinusoidal functions, a model that has been employed by a number of previous works (see the work by Meyer et al.^32^ for example). Sinusoidal functions are mathematically concise models which can naturally capture changes of environmental parameters in the nature, such as average temperature and daylength. We should note, however, that sinusoidal functions are symmetrical, i.e., positive and negative wave variations with respect to the baseline are identical, and thus their general applicability is not necessarily guaranteed. Other alternative modeling approaches, such as parabolas, could also be used for our analysis, which we believe would yield similar conclusions to what were drawn in this work. Regardless of these limitations, this study demonstrated robust patterns of seasonal variations of brain activity measured by resting-state fMRI, thus providing further evidence of a neurobiological impact of season on brain functions.

## Methods

### Data

MRI datasets used in this study were sourced from the HCP database (1200 Subjects Data Release: https://www.humanconnectome.org/study/hcp-young-adult), which includes 410 healthy young adults (60% female). The process for determining the sample size of the data was as follows: 1. Only data from the group with an age range of 26–35 years was selected to minimize age effects; 2. Data from acquisitions Q1-Q3 were excluded because they were reconstructed with an older algorithm. In addition, to ensure that the number of subjects is roughly the same in each acquisition quarter, we selected the last 8 consecutive quarters (two years) of data from Q6 to Q13; 3. Any data with QC_issues noted in the HCP quality control process were excluded. The data acquisition seasons for these subjects were defined as follows: Spring is from February 1st to April 30th; Summer is from May 1st to July 31st; Autumn is from August 1st to October 31st; and Winter is from November 1st to January 31st. The numbers of female subjects were 61, 57, 51, 78 for Spring, Summer, Autumn and Winter respectively, and the numbers of male subjects were 38, 34, 42, 49 for the four seasons respectively. It is noted that sex was regressed out from all the measures analyzed in this work to mitigate the influence of the nuisance variable. Resting-state fMRI images were used for analyzing seasonal variations of functional connectivity (FC) and network properties, which were acquired with multiband gradient‐echo echo‐planar imaging sequence with the following parameters: TR = 720 ms, TE = 33.1 ms, voxel size = 2 mm isotropic, number of volumes = 1200. Detailed MRI protocols are well described in a previous work^53^.

### Preprocessing

Functional MRI images from the HCP repository have been minimally preprocessed (see more details in a previous work^54^). Further processing of the fMRI images included regressing-out of nuisance variables from head movements, cardiac signals and respiratory signals using PhysIO toolbox^55^. Finally, time courses were bandpass filtered to retain the frequency from 0.01 to 0.1 Hz, and then normalized to unit variance voxel-wisely.

### Image analysis

#### Regions of interest

The basic FC analyses in this study were performed at a regional level. For GM, functional regions of interest (ROI) were defined using atlas from W. R. Shirer, et al.^40^. Meanwhile, WM was parcellated into fiber bundles using the JHU ICBM-DTI-81 WM atlas^41^. The preprocessed fMRI signals were averaged across voxels within each GM region and WM bundle, yielding region-averaged signals for subsequent analyses. Detailed WM bundles, GM regions (along with their abbreviations), and the list of all functional networks are presented in Supplementary Tables S1 and S2.

#### Power spectra of BOLD signals

The power spectra of region-averaged fMRI signals were computed for each WM bundle using the standard Welch’s method. In this work, the Welch’s power spectral density^56^ estimate function (pwelch) built in MATLAB was implemented. Note that the BOLD time course was not filtered in the power spectra computation. Based on the averaged periodogram, the fractional amplitude of low-frequency fluctuations (fALFF) was calculated as the ratio of average power across the frequency range of 0.01–0.08 Hz to the average power across all the frequencies higher than 0.01 Hz.

#### Functional connectivity matrices at global- and network-levels

WM-GM FC matrices were calculated based on Pearson correlation in region-averaged BOLD signals between each pair of WM-GM regions for each subject individually. Each matrix contained ${N}_{W}*{N}_{G}$ elements, with ${N}_{W}$ being the number of WM bundles and ${N}_{G}$ the number of GM regions. Fisher Z-Transform was applied to each FC matrix element-by-element, and the mean Z-score of each FC matrix was defined to be the average of absolute values of Z-scores over all its elements. In addition, WM-GM FC matrices were segmented into 14 submatrices across GM regions, representing 14 functional networks defined by Shirer et al.^40^, and mean Z-score of each submatrix was also computed by averaging across the absolute values of elements of each WM-GM submatrix. GM-GM and WM-WM matrices were also calculated, along with their corresponding mean Z-scores, similar to the WM-GM FC.

### Graph network analysis

Brain graph network was established based on GM–GM, WM–WM and GM–WM correlation matrices, which captured FC between each and every pair of functional regions throughout the brain. These matrices were thresholded at 0.2 and binarized for each subject, from which multiple network properties were derived group-wisely. The nodes of the graph are GM regions and WM bundles, and the edges between nodes are thresholded FC between them. The Brain Connectivity Toolbox^57^ (https://sites.google.com/site/bctnet/) was used to conduct the network analysis.

#### Density

Density is a relative measure of how many connections a network (graph) has. It is defined as the fraction of number of present connections to the number of possible connects in a graph:

1 $${{Density}} = \frac{{\# \;{{of}}\;{{present}}\;{{connections}}}}{{\# \;{{of}}\;{{possible}}\;{{connections}}}} = \frac{{\# \;{{of}}\;{{present}}\;{{connections}}}}{{\frac{1}{2}{{N}}\left( {{{N}} - 1} \right)}}$$

in which $N$ is the total number of nodes in the graph.

#### Transitivity

Transitivity, a.k.a. the global clustering coefficient, is a measure of the degree to which a graph is clustered. It is defined as the ratio of the number of all closed triplets to the total number of triplets including both closed and open triplets^58^. Assume $X$ is a connection matrix, the transitivity ${T}_{c}$ can be derived as:

2 $$T_{c} = \frac{{\sum\nolimits_{{i,j,k}} {X_{{ij}} } X_{{jk}} X_{{ki}} }}{{\sum\nolimits_{{i,j,k}} {X_{{ij}} } P_{{jk}} X_{{ki}} }}$$

where $P$ is the connection matrix with complete connections (i.e., an edge exists between any pair of distinct nodes). The above equation can be simplified as:

3 $$T_{c} = \frac{{tr\left( {X^{3} } \right)}}{{\sum\limits_{{i,j}} {\left( {X^{2} } \right)_{{ij}} } - tr\left( {X^{2} } \right)}}$$

#### Global efficiency

Global efficiency is a measure of how efficiently a network exchanges information through connections^59^. It is defined as the average of the inverse of the shortest distance between all pairs of nodes in a network:

4 $$E = \frac{1}{{N\left( {N - 1} \right)}}\sum\limits_{{i \ne j}} {\frac{1}{{d_{{ij}} }}}$$

where ${d}_{ij}$ is the shortest distance (i.e., minimum path length) between two nodes $i$ and $j$.

#### Characteristic path length

The characteristic path length measures, on average, the minimum number of edges through which one graph node reaches another. Assume the shortest path between any pair of graph nodes is computed, the characteristic path length is defined as the average of shortest paths between all possible pairs of nodes which are reachable from each other^60^.

### Seasonal variation analysis

Seasonal variations of FC and fALFF were modeled with the assumption that they fluctuate in a sinusoidal form with annual periodicity^32^:

5 $${y\left( t \right) = Acos\left( {\omega t + \vartheta } \right) + c }$$

where $y$ is the response variable, $\omega =2\pi /T$ ($T$ = 1 year), $\vartheta$ and $A$ are the phase and amplitude of the sinusoidal function, respectively. The above function can be expanded as:

6 $${y\left( t \right) = asin(\omega t) + bcos\left( {\omega t} \right) + c }$$

In this work, the period was mathematically set to 4 to reflect the four acquisition quarters. Specifically, $t$ = 0, 1, 2, and 3 for Winter, Spring, Summer and Autumn, respectively. Adjusted measures (after controlling for nuisance variable) of each subject were treated as one observation and F-test was used to determine the significance of seasonal periodicity.

To explore whether the seasonality effects were related to environmental factors, the two sinusoidal regressors were substituted by environmental factors for linear regression. The environmental factors included average daylength and temperature that were documented for St. Louis, Missouri during the time period studied. The *p* value for each coefficient was evaluated to determine the significance of the impact of each factor on the output measures.

### Supplementary Information

Supplementary Information.

## Data Availability

The dataset analyzed in this study is publicly available in the HCP database (1200 Subjects Data Release: https://www.humanconnectome.org/study/hcp-young-adult).
