# Supplementary material for: Seasonal variations of functional connectivity of human brains
Source: Sci Rep. 2023 Oct 6;13:16898. doi: 10.1038/s41598-023-43152-4 (PMC10558480; doi:10.1038/s41598-023-43152-4)
Supplement: Supplementary file 1 — Supplementary Information. [file 41598_2023_43152_MOESM1_ESM.pdf]

Supplementary Information for

Seasonal variations of functional connectivity of human brains

Lyuan Xu, Soyoung Choi, Yu Zhao, Muwei Li, Baxter P. Rogers, Adam Anderson, John C. Gore, Yurui Gao, Zhaohua Ding

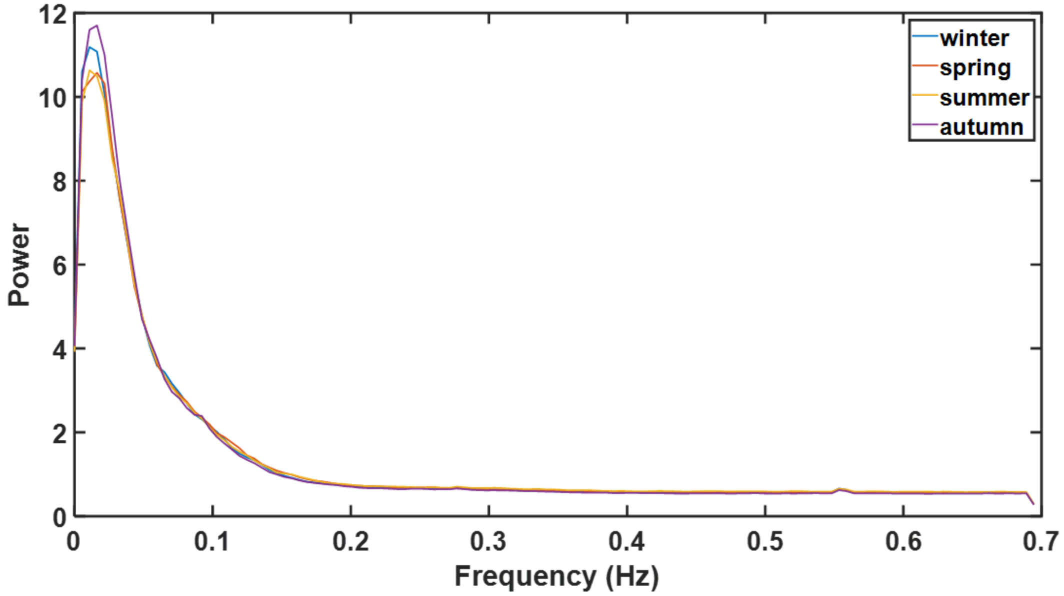

Figure S1. Mean power spectra of GM regions for four seasons. Each color denotes a season, and the autumn shows higher power in the frequency range of 0.01-0.08 Hz than other seasons.

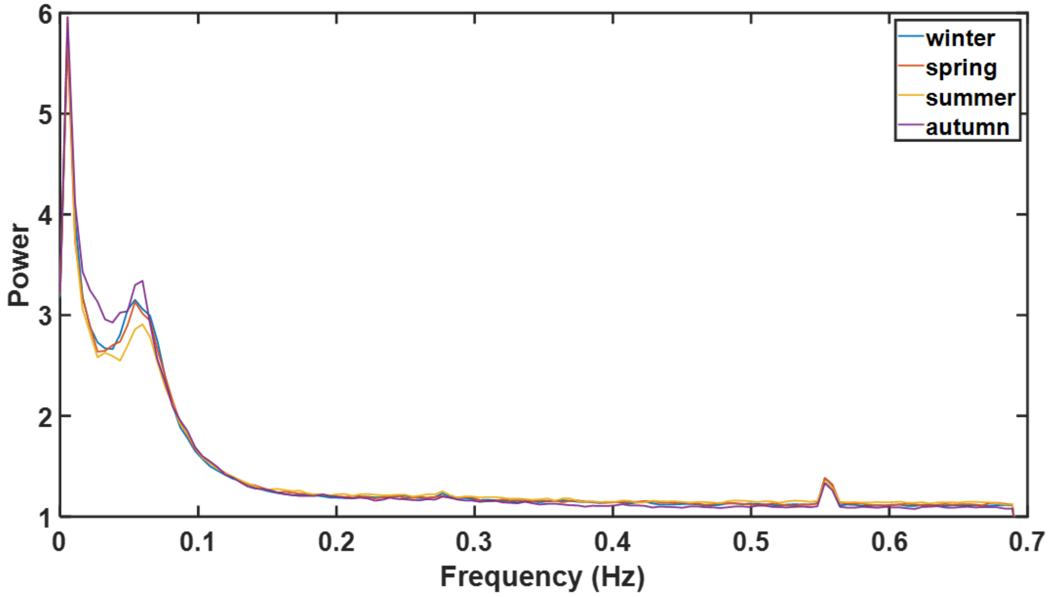

Figure S2. Mean power spectra of WM tracts for four seasons. Each color denotes a season, and the autumn shows higher power in the frequency range of 0.01-0.08 Hz than other seasons.

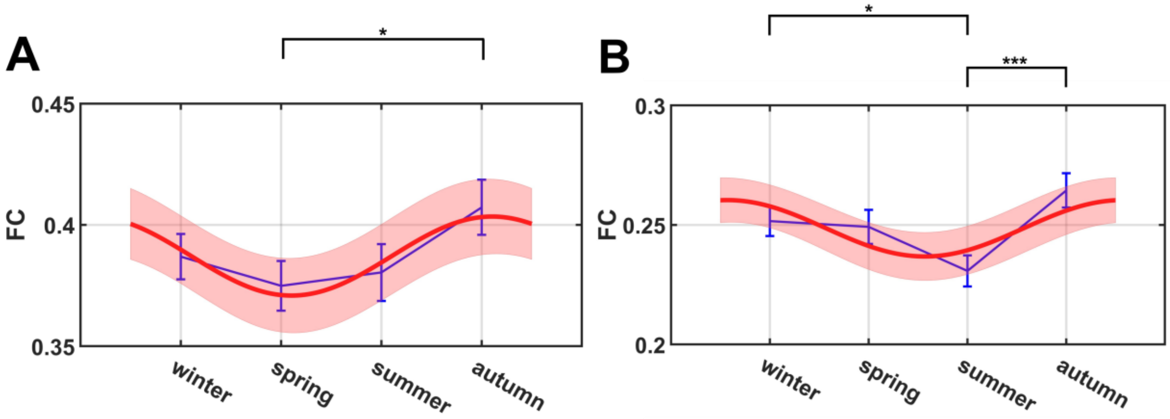

Figure S3. Group mean FC (blue) across seasons overlaid with proposed sinusoidal function fitting to test for periodicity and the related interval at 95% confidence level (red) separately for (A) GM-GM and (B) WM-WM FC matrices. The error bars (blue) are plotted based on the standard errors of the mean in each season. Note: * p<0.05; *** p<0.001.

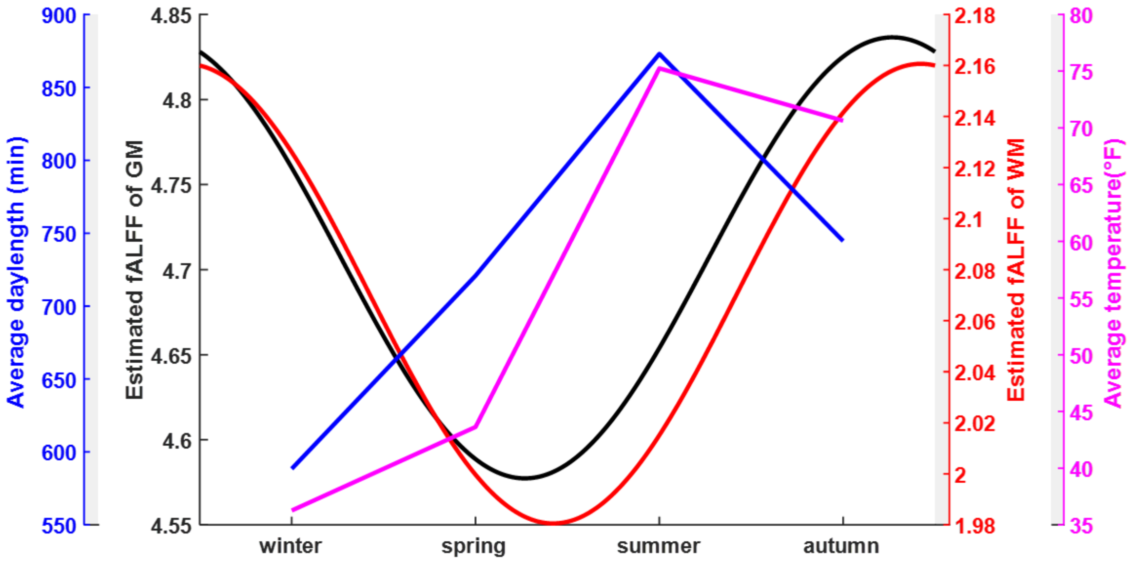

Figure S4. Estimates of mean fALFF of GM (black) and WM (red) and averaged seasonal environmental factors in St. Louis, MO across four seasons, including averaged temperature (°F, magneta) and averaged daylength (min, blue).

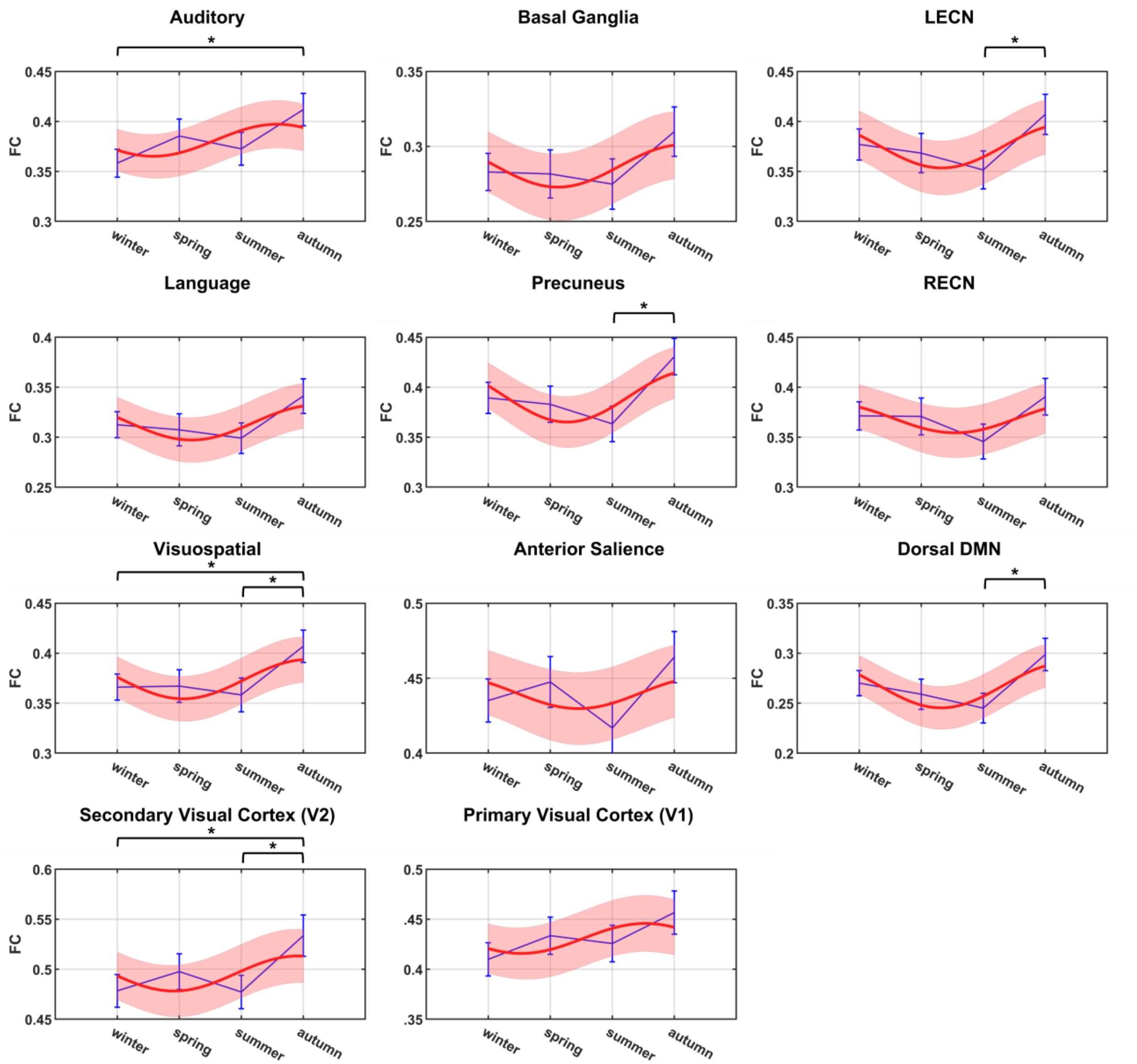

Figure S5. Group mean FC of brain networks (blue) across seasons overlayed with proposed sinusoidal function fitting to test for periodicity and the related interval at 95% confidence level (red) separately for other functional networks. The error bars (blue) were plotted based on the standard errors of the mean at each season. Note: * $p<0.05$; ** $p<0.01$.

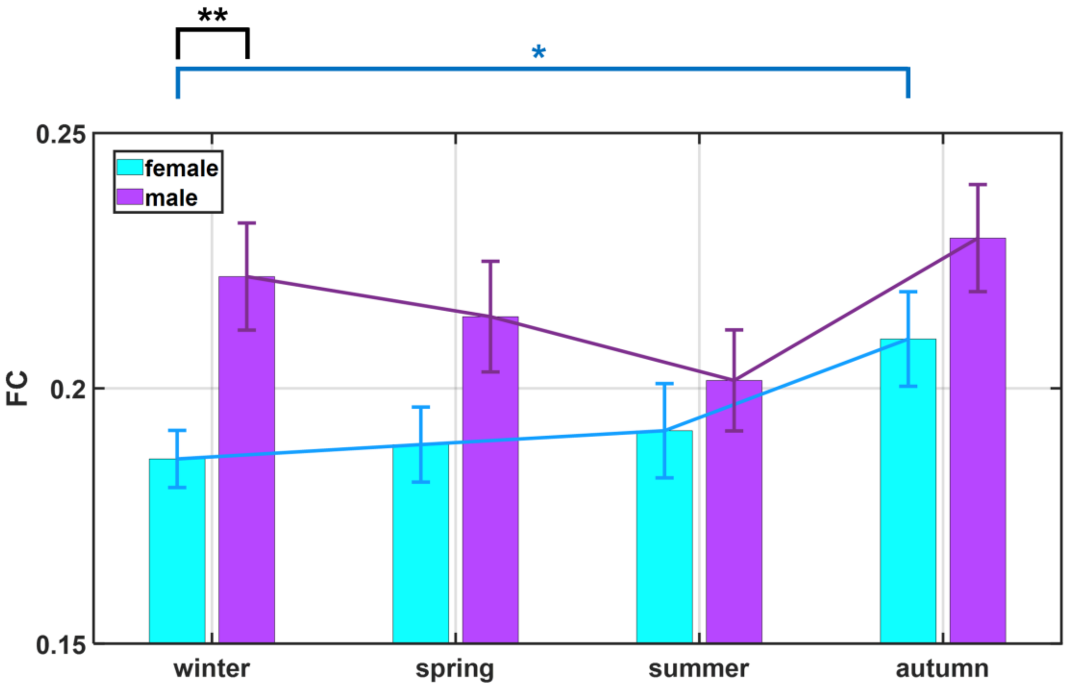

Figure S6. Seasonal variations in group mean of GM-WM FC matrices for female (cyan) and male (purple) subjects. The error bars are plotted based on the standard errors of the mean at each season. Note: * $p<0.05$; ** $p<0.01$.

Network	#ROI	Anatomical Location of Functional ROIs
Auditory	G1	Left Superior Temporal Gyrus, Heschl's Gyrus
	G2	Right Superior Temporal Gyrus
Basal Ganglia	G3	Left Thalamus, Caudate
	G4	Right Thalamus, Caudate, Putamen
	G5	Left Inferior Frontal Gyrus
	G6	Right Inferior Frontal Gyrus
Left Executive Control Network (LECN)	G7	Left Middle Frontal Gyrus, Superior Frontal Gyrus
	G8	Left Inferior Frontal Gyrus, Orbitofrontal Gyrus
	G9	Left Superior Parietal Gyrus, Inferior Parietal Gyrus, Precuneus, Angular Gyrus
	G10	Left Inferior Temporal Gyrus, Middle Temporal Gyrus
	G11	Right Crus I
Language	G12	Inferior Frontal Gyrus
	G13	Left Middle Temporal Gyrus
	G14	Left Middle Temporal Gyrus, Angular Gyrus
	G15	Left Middle Temporal Gyrus, Superior Temporal Gyrus, Supramarginal Gyrus, Angular Gyrus
	G16	Right Inferior Frontal Gyrus
	G17	Right Supramarginal Gyrus, Superior Temporal Gyrus, Middle Temporal Gyrus
	G18	Left Crus I
Precuneus	G19	Midcingulate Cortex, Posterior Cingulate Cortex
	G20	Precuneus
	G21	Left Angular Gyrus
	G22	Right Angular Gyrus
Right Executive Control Network (RECN)	G23	Right Middle Frontal Gyrus, Right Superior Frontal Gyrus
	G24	Right Middle Frontal Gyrus
	G25	Right Inferior Parietal Gyrus, Supramarginal Gyrus, Angular Gyrus
	G26	Right Superior Frontal Gyrus
	G27	Left Crus I, Crus II, Lobule VI
	G28	Right Caudate
Sensorimotor	G29	Left Precentral Gyrus, Postcentral Gyrus
	G30	Right Precentral Gyrus, Postcentral Gyrus
	G31	Right Supplementary Motor Area
	G32	Bilateral Lobule IV, Lobule V, Lobule VI
Visuospatial	G33	Left Middle Frontal Gyrus, Superior Frontal Gyrus, Precentral Gyrus
	G34	Left Inferior Parietal Sulcus
	G35	Left Frontal Operculum, Inferior Frontal Gyrus
	G36	Left Inferior Temporal Gyrus
	G37	Right Middle Frontal Gyrus
	G38	Right Inferior Parietal Lobule
	G39	Right Frontal Operculum, Inferior Frontal Gyrus
	G40	Right Middle Temporal Gyrus
	G41	Left Lobule VIII, Lobule VIIb
	G42	Right Lobule VIII, Lobule VIIb
	G43	Right Lobule VI, Crus I
Anterior Salience	G44	Left Middle Frontal Gyrus
	G45	Left Insula
	G46	Anterior Cingulate Cortex, Medial Prefrontal Cortex, Supplementary Motor Area
	G47	Right Middle Frontal Gyrus
	G48	Right Insula
	G49	Left Lobule VI, Crus I
	G50	Right Lobule VI, Crus I
Dorsal DMN	G51	Medial Prefrontal Cortex, Anterior Cingulate Cortex, Orbitofrontal Cortex
	G52	Left Angular Gyrus
	G53	Right Superior Frontal Gyrus
	G54	Posterior Cingulate Cortex, Precuneus
	G55	Midcingulate Cortex
	G56	Right Angular Gyrus
	G57	Left and Right Thalamus
	G58	Left Hippocampus
	G59	Right Hippocampus
Secondary Visual Cortex (V2)	G60	Left Middle Occipital Gyrus, Superior Occipital Gyrus
	G61	Right Middle Occipital Gyrus, Superior Occipital Gyrus
Posterior Insula	G62	Left Middle Frontal Gyrus
	G63	Left Supramarginal Gyrus, Inferior Parietal Gyrus
	G64	Left Precuneus
	G65	Right Midcingulate Cortex
	G66	Right Superior Parietal Gyrus, Precuneus
	G67	Right Supramarginal Gyrus, Inferior Parietal Gyrus
	G68	Lobule VI
	G69	Left Posterior Insula, Putamen
	G70	Lobule VI
	G71	Right Posterior Insula
Primary Visual Cortex (V1)	G72	Calcarine Sulcus
Ventral DMN	G73	Left Retrosplenial Cortex, Posterior Cingulate Cortex
	G74	Left Middle Frontal Gyrus
	G75	Left Parahippocampal Gyrus
	G76	Left Middle Occipital Gyrus
	G77	Right Retrosplenial Cortex, Posterior Cingulate Cortex
	G78	Precuneus
	G79	Right Superior Frontal Gyrus, Middle Frontal Gyrus
	G80	Right Parahippocampal Gyrus
	G81	Right Angular Gyrus, Middle Occipital Gyrus
	G82	Right Lobule IX

Table S1. List of GM ROIs and corresponding functional networks.

WM tract	WM tract description
MCBP	middle cerebellar peduncle
GCC	genu of corpus callosum
BCC	body of corpus callosum
SCC	splenium of corpus callosum
FX	fornix
CST_(L,R)	corticospinal tract (left, right)
ML_(L,R)	medial lemniscus (left, right)
ICBP_(L,R)	inferior cerebellar peduncle (left, right)
SCBP_(L,R)	superior cerebellar peduncle (left, right)
CP_(L,R)	cerebral peduncle (left, right)
ALIC_(L,R)	anterior limb of internal capsule (left, right)
PLIC_(L,R)	posterior limb of internal capsule (left, right)
RLIC_(L,R)	retrolenticular part of the internal capsule (left, right)
ACR_(L,R)	anterior corona radiata (left, right)
SCR_(L,R)	superior corona radiata (left, right)
PCR_(L,R)	posterior corona radiata (left, right)
PTR_(L,R)	posterior thalamic radiation (left, right)
SS_(L,R)	sagittal stratum (left, right)
EC_(L,R)	external capsule (left, right)
CGG_(L,R)	cingulum in the cingulate gyrus (left, right)
CGH_(L,R)	cingulum in the hippocampus (left, right)
FXC_(L,R)	fornix (cres) (left, right)
SLF_(L,R)	superior longitudinal fasciculus (left, right)
SFO_(L,R)	superior fronto-occipital fasciculus (left, right)
UF_(L,R)	uncinate fasciculus (left, right)
TAP_(L,R)	Tapetum (left, right)

Table S2. List of WM bundles.
